# Wind Turbine Blade Fault Diagnosis Integrating Multi-Scale Enhanced Hierarchical Fuzzy Entropy, Isolation Forest and GWO-GRU

**DOI:** 10.3390/e28070810

**Published:** 2026-07-16

**Authors:** Min Wang, Xiao-Fei Zhang, Guo-Jun Qin, Ming Liu

**Affiliations:** 1 School of Engineering Science, Shandong Xiehe University, Jinan 250107, China; qgjnudt@163.com; 2 College of Electrical and Information Engineering, Hunan University, Changsha 410072, China; zhangxiaofei@hnu.edu.cn; 3 SinoAzure Wind Power Co., Ltd., Xiangtan 411101, China; liuming@hewp.com.cn

**Keywords:** wind turbine blade, fault diagnosis, MEHFE, Isolation Forest, GRU, GWO

## Abstract

To effectively extract fault characteristics from complex vibration signals and improve the diagnostic performance of deep learning networks, this paper introduces a wind turbine blade fault diagnosis method that combines Multi-scale Enhanced Hierarchical Fuzzy Entropy (MEHFE), Isolation Forest, and the Grey Wolf Optimization (GWO) algorithm for optimizing the Gated Recurrent Unit (GRU). Initially, the MEHFE algorithm is applied to decompose and reconstruct three-directional vibration signals at the blade root, thereby extracting “scale-frequency” dual-dimensional features that represent the evolution of fault frequency structure and complexity across multiple scales. Subsequently, Isolation Forest is employed to assess and filter feature importance, constructing an optimal feature subset to mitigate redundancy and noise interference. Finally, the optimal features are fed into the GRU network for fault pattern recognition, and the GWO algorithm is utilized to adaptively optimize network hyperparameters, thereby enhancing classification accuracy and noise resilience. Simulation experiments on typical wind turbine blade faults reveal that when GRU serves as the classifier, the diagnostic accuracy of MEHFE exceeds 76%. After feature optimization with Isolation Forest and network parameter optimization with GWO, the diagnostic accuracy surpasses 93%, demonstrating notable advantages in both classification capability and stability. Even under conditions of noise interference, the accuracy remains above 90%. The research substantiates that the proposed method can effectively extract pattern information indicative of blade structural damage from vibration data, achieving high fault recognition accuracy and robustness.

## 1. Introduction

As the wind power industry undergoes large-scale expansion, both the quantity of wind turbines and their installed capacity continue to rise, with malfunctions and structural damage issues becoming increasingly pronounced [1]. Wind turbine failures not only result in reduced power generation efficiency but also substantially escalate operation and maintenance expenses, leading to a loss of power generation capacity. If not promptly identified and rectified, these failures may even precipitate safety incidents, posing grave risks to personnel and equipment [2,3]. Consequently, the safety and reliability concerns in wind energy utilization have garnered widespread attention. As a pivotal component of wind turbines, the performance and reliability of blades directly influence the overall system’s efficiency and safety. Moreover, blades exhibit a relatively high failure rate and incur significant maintenance costs [4,5]. Hence, conducting research on fault diagnosis methods for wind turbine blades holds substantial engineering application value and practical significance.

At present, the main approaches for fault diagnosis in wind turbine blades encompass acoustic emission technology [6,7], infrared thermal imaging inspection [8,9], image recognition technology [10,11], non-contact acoustic inspection [12,13], and vibration detection methodology [14,15]. Among these approaches, acoustic emission technology demonstrates a high degree of sensitivity towards micro-cracks; however, its performance is prone to interference from ambient noise. Infrared thermal imaging enables the visual identification of anomalies within the structural temperature field, yet this technique necessitates external thermal excitation and exhibits limitations in the detection of deep-seated defects. Image recognition facilitates visual inspections, but its efficacy is markedly influenced by variations in illumination and surface contamination, posing challenges in accurately discerning internal damage. Non-contact acoustic detection, despite its operational flexibility, is susceptible to interference from wind noise and exhibits inadequate responsiveness to low-frequency signals. In contrast, the vibration detection method, grounded in structural dynamics theory, accomplishes damage identification by inversely deriving anomalies in system parameters through vibration response analysis [16,17]. This method offers several advantages, including straightforward sensor installation, the capability for online continuous monitoring, sensitivity to alterations in local stiffness, and the provision of abundant information [18]. Vibration signal analysis effectively extracts early fault indicators from both blades and root bearings, thereby establishing itself as a predominant technical approach in the realm of wind turbine blade damage monitoring.

The diagnostic efficacy of vibration detection methods hinges critically on the precision of feature extraction, thereby rendering the optimization of feature engineering pivotal for augmenting fault identification capabilities. Nevertheless, the intricate operational conditions and severe service environments of wind turbine blades result in vibration signals that demonstrate pronounced non-linearity, non-stationary, and a low signal-to-noise ratio. Traditional signal processing techniques, encompassing time-domain analysis [19], frequency-domain analysis [20], time-frequency analysis [21], wavelet analysis [22], and modal decomposition [23], struggle to effectively extract fault features characterized by sensitivity and discernibility, thus exhibiting notable constraints in practical engineering applications [24,25].

In 1948, Shannon incorporated the concept of entropy into information theory [26]. As a statistical metric, entropy enables the quantification of system complexity and the monitoring of dynamic changes through the analysis of the nonlinear behavior inherent in time series. In comparison to conventional signal processing techniques, feature extraction methods grounded in entropy theory demonstrate superior classification accuracy and enhanced pattern recognition capabilities [27,28]. Consequently, subsequent to Shannon entropy, a variety of other entropies, including Rayleigh Entropy (REn) [29], Approximate Entropy (ApEn) [30], Sample Entropy (SampEn) [31], Permutation Entropy (PE) [32], and Fuzzy Entropy (FE) [33], have been progressively introduced and extensively utilized in the realm of mechanical equipment fault diagnosis, emerging as pivotal tools for vibration signal analysis and fault feature extraction [34,35]. Among these, FE, serving as an indicator for assessing the complexity of time series, exhibits robust noise resistance properties and finds widespread application in fault diagnosis engineering for components like bearings and gears.

To address the challenge that basic scale entropy fails to effectively evaluate time series across multiple time scales, Costa et al. [36] proposed the multi-scale entropy method. Subsequently, Zheng Jinde et al. [37] further developed the Multi-scale Fuzzy Entropy (MFE) algorithm and applied it to the fault diagnosis of rolling bearings. Nevertheless, the coarse-graining process utilized in this algorithm inevitably leads to the loss of high-frequency information during the processing of the original signal, thereby hindering a comprehensive representation of the complete pattern information inherent in the original signal. To surmount this limitation, Song Yedong et al. [38] integrated the multi-level analysis method [39] with FE to introduce the Enhanced Hierarchical Fuzzy Entropy (EHFE) algorithm, which has been successfully employed in diesel engine fault diagnosis. In contrast to the “coarse-graining” process in the multi-scale entropy method, the enhanced hierarchical analysis method can effectively extract both high- and low-frequency components from the original signal. However, it is still constrained to a single original scale and cannot capture the dynamic variations of these frequency components as the time scale expands.

To address the aforementioned issues, this research introduces a fault diagnosis method for wind turbine blades that combines Multi-scale Enhanced Hierarchical Fuzzy Entropy (MEHFE), Isolation Forest, and the Grey Wolf Optimization algorithm to optimize the Gated Recurrent Unit (GWO-GRU). The primary innovations are outlined as follows:

(1) The MEHFE algorithm is proposed, integrating multi-scale decomposition with enhanced hierarchical analysis to dissect vibration signals across the dual dimensions of “scale-frequency”. This approach surmounts the constraints of traditional entropy features, which are confined to a single-dimensional description, thereby offering a more comprehensive depiction of blade damage patterns.
(2) Isolation Forest is employed to assess and filter the significance of high-dimensional features within MEHFE, facilitating the construction of an optimal feature subset. This effectively mitigates redundancy and noise interference, enhancing both diagnostic efficiency and feature robustness.
(3) By utilizing GRU as the classifier and integrating the GWO algorithm to adaptively refine its hyper-parameters, a GWO-GRU model is established. This model circumvents the uncertainties inherent in manual parameter tuning and bolsters classification accuracy and generalization capabilities.
(4) The three components are seamlessly integrated to forge a complete diagnostic chain encompassing “feature extraction—feature selection—parameter optimization—pattern recognition”, thereby presenting a systematic approach to wind turbine blade fault diagnosis.

## 2. Methodology

### 2.1. MEHFE Algorithm

#### 2.1.1. Fuzzy Entropy

Sample entropy utilizes a step function to assess the similarity between vectors. However, this rigid categorization overlooks the boundary ambiguity inherent in practical applications, thereby failing to adequately capture the true similarity relationships between vectors. To tackle this problem, Chen et al. [31] introduced the FE algorithm that employs a fuzzy function capable of addressing fuzzy boundaries to evaluate vector similarity [40]:

Step 1: Given a one-dimensional data $X=\{x_{1},x_{2},\cdots x_{i},\cdots ,x_{N}\}$ with length N, convert it into an *m*-dimensional vector $Y_{i}^{m}$:

(1) $$\begin{array}{cc} Y_{i}^{m}=[x_{i},x_{i+1},\cdots ,x_{i+m-1}]-u_{i} & \left(i=1,2,\cdots ,N-m+1\right) \end{array}$$

where $u_{i}=\frac{1}{m}\sum_{j=0}^{m-1}x_{i+j}$ represents the local baseline of the *i*-th reconstructed vector.

Step 2: Calculate the Chebyshev distance $d_{i,j}^{m}$ between two adjacent *m*-dimensional vectors in $Y_{i}^{m}$:

(2) $$\begin{array}{c} d_{i,j}^{m}=d\left[Y_{i}^{m},Y_{j}^{m}\right]=\underset{k}{\max}\left[\left|Y_{i+k}^{m}-Y_{j+k}^{m}\right|\right],\;k=0,1,\cdots ,N-m \end{array}$$

Step 3: Introduce the fuzzy function $A_{i,j}^{m}$:

(3) $$A_{i,j}^{m}=\exp\left[-{\left(\frac{d_{i,j}^{m}}{r}\right)}^{n}\right]$$

where r represents the similarity tolerance parameter, typically set to r=0.1~0.25SD, where *SD* is the standard deviation of the original data X, and n is the gradient of the fuzzy function, typically set to n=2.

Step 4: Calculate the fuzzy similarity between two adjacent *m*-dimensional vectors $\phi^{m}(r)$:

(4) $$\phi^{m}(r)=\frac{1}{N-m}\sum_{i=1}^{N-m}\left(\frac{1}{N-M-1}\sum_{j=1,j\neq i}^{N-m}A_{i,j}^{m}\right)$$

Step 5: Convert X into an m+1-dimensional vector $Y_{i}^{m+1}$, repeat steps 1 to 4, and calculate $\phi^{m+1}(r)$;

Step 6: Calculate FE:

(5) $$FE[X,m,n,r]=\ln\left[\frac{\phi^{m}(r)}{\phi^{m+1}(r)}\right]$$

#### 2.1.2. Multi-Scale Fuzzy Entropy

The basic scale FE algorithm struggles to comprehensively represent the information-laden characteristics of real-world signals. To overcome this limitation, Zheng Jinde et al. [37] integrated the multi-scale method [36] into FE and proposed the MFE algorithm:

Step 1: For one-dimensional data X, calculate the multiscale time series $y_{j}^{\tau }$:

(6) $$\begin{array}{cc} y_{j}^{\tau }=\frac{1}{\tau }\sum_{i=(j-1)\tau +1}^{j\tau }x_{i} & \left(1\leq j\leq \frac{N}{\tau }\right) \end{array}$$

where τ is the scale factor.

Step 2: Calculate the FE of τ time series separately to obtain the MFE of X:

(7) $$\begin{array}{c} MFE[X,m,n,r,\tau ]=\left\{FE[y_{j}^{l},m,n,r]\right\},\;l=1,2,\cdots ,\tau \end{array}$$

#### 2.1.3. Enhanced Hierarchical Fuzzy Entropy

The MFE algorithm fundamentally represents a “coarse-graining” analytical procedure grounded in sliding averages, a process that is prone to causing the loss of high-frequency information within the original time series during analysis. To effectively extract both high- and low-frequency components from the original signal, Song Liye et al. [38] introduced the EHFE algorithm by integrating enhanced hierarchical analysis [39] with FE:

Step 1: For one-dimensional data X, define high-frequency and low-frequency components $Q_{1}(x)$ and $Q_{0}(x)$:

(8) $$\left\{\begin{array}{cc} \begin{array}{c} Q_{0}(x)=\frac{x_{i}+x_{i+1}}{2}\\ Q_{1}(x)=\frac{x_{i}-x_{i+1}}{2} \end{array}, & i=1,2,\cdots ,N-1 \end{array}\right.$$

The matrix representation of the high-low frequency operator $Q_{t}^{s}$ (t=0 or t=1) corresponding to the *s*-th layer is as follows:

(9) $$Q_{l}^{s}={\left[\begin{array}{cccccccc} \frac{1}{2} & \underset{2^{s-1}-1}{\underset{︸}{0\cdots 0}} & \frac{{\left(-1\right)}^{t}}{2} & 0 & \cdots & 0 & 0 & 0\\ 0 & \frac{1}{2} & \underset{2^{s-1}-1}{\underset{︸}{0\cdots 0}} & \frac{{\left(-1\right)}^{t}}{2} & \cdots & 0 & 0 & 0\\ \vdots & \vdots & \vdots & \vdots & \ddots & \vdots & \vdots & \vdots \\ 0 & 0 & 0 & 0 & \cdots & \frac{1}{2} & \underset{2^{s-1}-1}{\underset{︸}{0\cdots 0}} & \frac{{\left(-1\right)}^{t}}{2} \end{array}\right]}_{\left(N-2^{s}+1\right)\times \left(N-2^{s-1}+1\right)}$$

Step 2: Construct a vector $V=\left[v_{1},v_{2},\cdots ,v_{s}\right]$, where $v_{s}\in \{0,1\}$, then the integer e can be expressed as:

(10) $$e=\sum_{j=1}^{s}2^{s-j}v_{j}$$

where the elements in e and V correspond one-to-one.

Step 3: Based on vector V, the components of each node in the *s*-th layer of the original signal X under the enhanced hierarchical analysis are defined as follows:

(11) $$X_{s,e}=Q_{v_{s}}^{s}Q_{v_{s-1}}^{s-1}\cdots Q_{v_{1}}^{1}X$$

where $X_{s,e}$ represents the *i*-th node component of the *s*-level decomposition, and $e=1,2,\cdots ,2^{s}$.

Step 4: Calculate the FE of the components of each node in the *s*-th layer, obtaining $2^{s}$ entropy values. The EHFE of X is defined as:

(12) $$\begin{array}{c} EHFE(X,m,n,r,s)=\left\{FE\left[X_{k,e},m,n,r\right]\right\},\;e=1,2,\cdots ,2^{s} \end{array}$$

#### 2.1.4. Multi-Scale Enhanced Hierarchical Fuzzy Entropy

Although EHFE addresses the limitation of MFE—namely, its inability to differentiate between frequency levels while simultaneously extracting both high and low-frequency components of a signal—it remains confined to a single original scale and fails to capture the dynamic variations in these frequency components as the time scale expands. To tackle this challenge, this research organically combines enhanced hierarchical decomposition with multi-scale analysis, introduces a MEHFE algorithm, and establishes a two-dimensional joint “scale-frequency” analysis framework. This framework facilitates joint analysis across two dimensions: in the scale dimension, it enables observation of the evolutionary process of low-frequency trends as the degree of coarse granulation intensifies; in the frequency dimension, it allows tracking of the attenuation trajectory of high-frequency details following progressive smoothing. The two-dimensional complexity features derived through this approach provide a more comprehensive and discriminative set of modal information for the identification and analysis of structural damage in wind turbine blades.

The calculation process of MEHFE is as follows:

Step 1: Perform multi-scale coarse graining on one-dimensional data X to obtain coarse graining sequences under different scale factors;

Step 2: Perform hierarchical decomposition on each coarse-grained sequence to obtain high-frequency and low-frequency sub-sequences for each node in the *s*-level decomposition;

Step 3: Calculate FE for the sub-sequence of each node;

Step 4: By integrating the FE of each node sub-sequence in each coarse-grained sequence, a two-dimensional feature vector is constructed, representing the MEHFE:

(13) $$\begin{array}{c} MEHFE(X,m,n,r,\tau ,s)={\left\{FE\left[{\left(X_{s,e}\right)}_{j}^{l},m,n,r\right]\right\}}_{\tau \times 2^{s}},\;l=1,2,\cdots ,\tau ;e=1,2,\cdots ,2^{s} \end{array}$$

### 2.2. Feature Selection Utilizing Isolation Forest

Within the framework of MEHFE, which combines multi-scale coarse granulation with multi-level high-low frequency decomposition, the ultimately generated feature vectors can possess dimensions numbering in the tens or even hundreds. For instance, when τ=8 and k=3, the feature information will attain 64 dimensions, inevitably encompassing a substantial amount of redundant information and noise features introduced by unreliable short-term sequence estimation. These detrimental factors can impede the precise identification and classification of wind turbine blade faults. Consequently, it becomes imperative to conduct dimensionality reduction on the high-dimensional feature set to eliminate irrelevant and redundant components, mitigate the “curse of dimensionality,” and thereby enhance the generalization capability and computational efficiency of the classifier. More crucially, dimensionality reduction can distill the essential from the non-essential, selecting core features that are genuinely sensitive to scale variations and frequency levels, thereby unveiling the fault evolution patterns concealed within complex vibration data.

Isolation Forest is an unsupervised anomaly detection algorithm grounded in ensemble learning. It builds multiple isolation trees through the random partitioning of the feature space and computes the mean path length of samples across these trees. Following normalization, anomaly scores are derived to ascertain whether a sample constitutes an outlier. The algorithm boasts several merits, including low computational complexity, the absence of a requirement for distance metrics, and robustness when dealing with high-dimensional data, thereby rendering it apt for large-scale anomaly detection tasks [41,42]. Although Isolation Forest is not directly applicable to feature selection, the significance of each feature can be appraised by tallying the frequency at which it is chosen as a splitting feature across all isolation trees, thus facilitating feature dimensionality reduction. The procedural steps for feature selection based on Isolation Forest are delineated as follows:

Step 1: Construct an isolation forest. Select ψ samples randomly from the dataset, recursively construct T isolated trees, and allow each tree to grow to a height limit of $h_{\max}=\left\lceil \log_{2}\psi \right\rceil$, where $\left\lceil \cdot \right\rceil$ denotes the ceiling function;

Step 2: Record splitting features. During the construction process of each tree, whenever an internal node randomly selects a feature for splitting, record one “use” of that feature;

Step 3: Count the number of selections. For all T trees, calculate the total number of selections $C_{i}$ for each feature i;

Step 4: Calculate the importance score $I_{i}$ for each feature, which represents the proportion of times when feature i is selected in all isolated tree splitting operations:

(14) $$I_{i}=\frac{C_{i}}{\sum_{i=1}^{D}C_{i}}$$

where D is the total number of features.

Step 5: Sort the D values of $I_{i}$ in descending order and select the top K features to form the optimal feature subset.

### 2.3. GWO-GRU

#### 2.3.1. Gated Recurrent Unit

**Figure 1:**
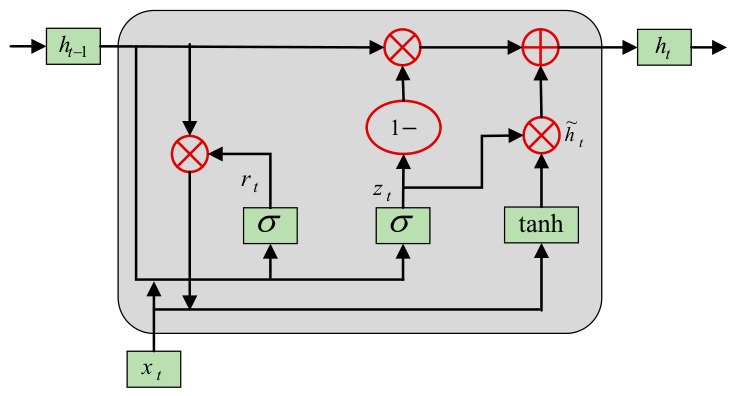
Structure diagram of GRU neuron.

The GRU neuron comprises an update gate and a reset gate, as depicted in Figure 1. Specifically, the update gate modulates the transmission intensity of historical information, governing the degree to which the previous time step’s state carries over to the current time step. Conversely, the reset gate regulates the proportion of historical information integrated with the current input, where a lower value signifies a greater extent of forgetting past information. This streamlined yet effective design allows GRU to preserve its capacity for long-term dependency modeling while substantially enhancing model training efficiency [43,44].

At time t, the internal state of the GRU neuron is represented as follows:

(15) $$r_{t}=\sigma \left(U_{r}x_{t}+W_{r}h_{t-1}+b_{r}\right)$$

(16) $$z_{t}=\sigma \left(U_{z}x_{t}+W_{z}h_{t-1}+b_{z}\right)$$

(17) $$h_{t}=\tanh\left(U_{h}x_{t}+W_{h}\left(r_{t}⊗h_{t-1}\right)+b_{h}\right)$$

(18) $$h_{t}=\left(1-z_{t}\right)⊗h_{t-1}+z_{t}⊗h_{t}$$

where $x_{t}$ is the input data at time t; $r_{t}$ and $z_{t}$ are the outputs of the reset gate and update gate respectively; $h_{t}$ is the hidden state; U, W, and b are the weight and bias matrices respectively; σ is the Sigmoid activation function, and ${\tilde{h}}_{t}$ is the temporary hidden state.

#### 2.3.2. Grey Wolf Optimization Algorithm

GWO is an intelligent optimization algorithm inspired by the social hierarchy and hunting behavior of gray wolf packs. It categorizes the wolf pack into four ranks: α, β, δ and ω, representing the current optimal, sub-optimal, third-optimal, and ordinary individuals, respectively. The optimization process of the algorithm centers on hunting behavior, and primarily comprises three stages [45,46]:

(1) Tracking and Encirclement: The wolf pack gradually closes in on the prey by moving, forming an encirclement progressively. At this juncture, the algorithm undertakes global exploration to identify potential optimal regions within the solution space.
(2) Enclosure Contraction: As iterations advance, the wolf pack progressively tightens its encirclement, converging towards the current optimal individual, and the algorithm transitions from global exploration to localized, refined search.
(3) Attacking Prey: Ultimately, the wolf pack converges on the prey’s location, and the algorithm correspondingly converges near the current optimal solution, thereby concluding the optimization process.

#### 2.3.3. Procedure of GWO Optimizing GRU

Hyper-parameters exert a decisive influence on the training dynamics, convergence rate, performance, and generalization capability of deep learning networks [47]. When employing GRU network as a classifier, the number of neurons in the hidden layer directly dictates the model’s memory capacity: an insufficient number can readily induce under-fitting, whereas an excessive number not only precipitates over-fitting but also markedly diminishes convergence efficiency. Although an overabundance of neurons in the fully connected layer can expedite the model’s fitting process during the initial training phase, it is prone to triggering over-fitting issues in the subsequent stages. The dropout rate, recognized as the most straightforward and efficacious regularization technique, effectively enhances generalization capability by compelling the model to assimilate redundant features. Nevertheless, an excessively high dropout rate can constrain model capacity, resulting in sluggish training progression and under-fitting. The learning rate concurrently impacts convergence rate and ultimate performance: an overly large value can instigate severe oscillations or even divergence throughout the training process, while an excessively small value can lead to exceedingly sluggish convergence and a propensity to become ensnared in local optima. To circumvent the uncertainties associated with manual parameter selection, in this research, GWO is employed to perform adaptive optimization of the hyper-parameters of the GRU network. The algorithm procedure is delineated as follows:

Step 1: Given the training set and test set;

Step 2: Initialization parameters, including population size, maximum iteration count, gray wolf control parameters, number of neurons in the hidden layer, number of neurons in the fully connected layer, dropout rate, and search range for learning rate;

Step 3: Randomly generate the locations of gray wolf populations, construct a GRU classifier with recognition accuracy as the fitness function, and calculate the network diagnosis accuracy under the initial parameters;

Step 4: Sort all gray wolves in descending order based on recognition accuracy, and label the top three individuals as wolves α, β, and δ, representing the three currently optimal combinations of parameters;

Step 5: Based on the position information of wolves α, β, and δ, guide each gray wolf to update its own position and generate a new position vector;

Step 6: Update the hyper-parameters of the GRU network based on the position of wolf α, and calculate the recognition accuracy of the model;

Step 7: Repeat step 4 to step 6 until the maximum number of iterations is reached;

Step 8: Output the globally optimal hyper-parameters and construct a GRU classifier. Employ the stochastic gradient descent algorithm for end-to-end training to obtain the network weights and biases.

## 3. The Framework of the Proposed Method

**Figure 2:**
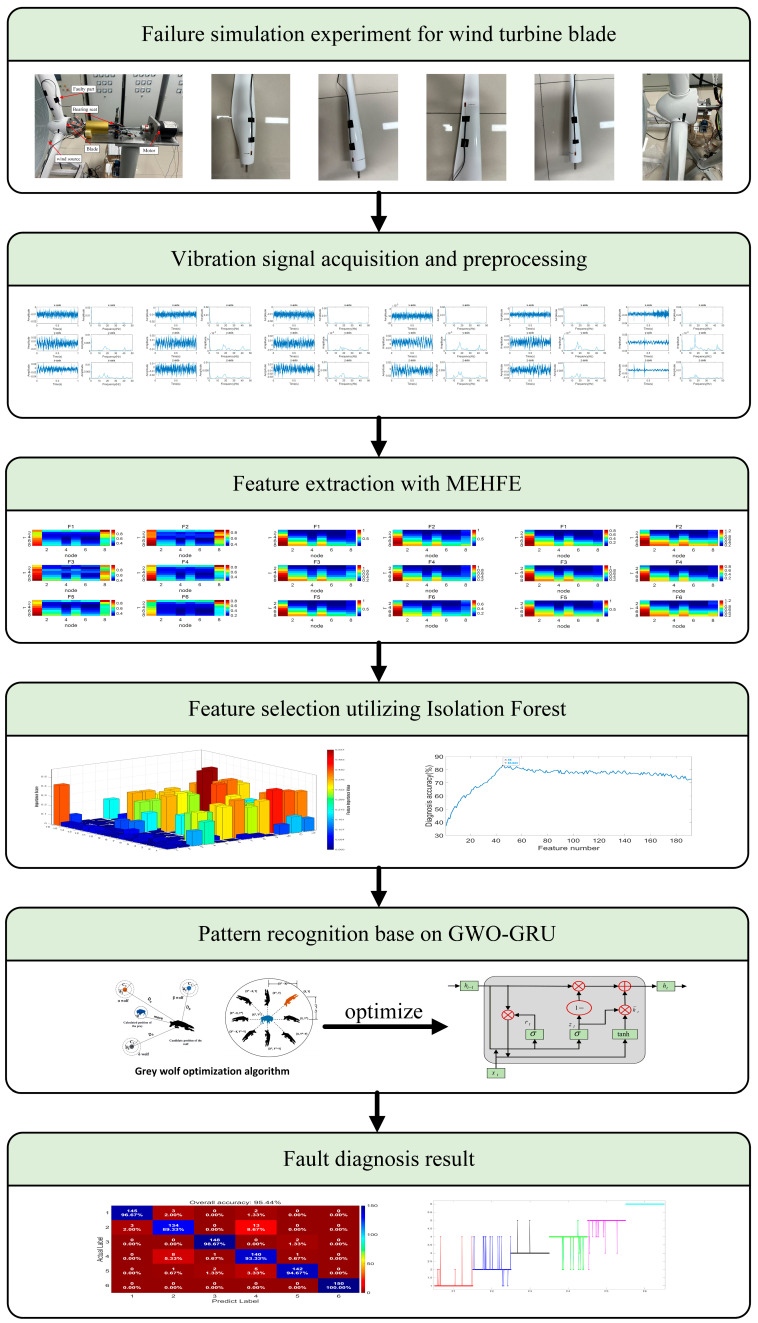
The framework of the fault diagnosis method integrating MEHFE, Isolation Forest and GWO-GRU.

In this research, a fault diagnosis method for wind turbine blades that integrates MEFHF, Isolation Forest and GWO-GRU is proposed. The framework of the method is shown in Figure 2, and the main steps of the method are as follows:

Step 1: Acquire three-directional vibration signals from the blade root bearing housing of wind turbines under various typical fault modes and different operational conditions using a data acquisition device, thereby providing the foundational raw data for subsequent feature extraction.

Step 2: Establish appropriate analysis scale ranges and hierarchical decomposition depths, compute the MEHFE for each of the three-directional vibration signals separately, extract “scale-frequency” dual-dimensional feature information capable of simultaneously representing the frequency structure variations and complexity evolution of faults under multi-scale conditions, and construct an initial high-dimensional feature space.

Step 3: Evaluate the significance of each feature within the “scale-frequency” two-dimensional analytical framework constructed by MEHFE, based on the frequency at which each feature is selected as an anomaly-splitting feature in the Isolation Forest algorithm. According to the importance ranking, eliminate redundant and interfering features, and select the optimal feature subset with the highest discriminatory power for subsequent fault diagnosis modeling.

Step 4: Allocate the optimal feature subset obtained in Step 3 into training and test sets in a certain proportion. Employ the GWO algorithm, utilizing the recognition accuracy of the GRU network on the validation set as the fitness function, to conduct a global optimization of its key hyper-parameters and search for the optimal hyper-parameter combination.

Step 5: Construct a GRU classifier model based on the optimal hyperparameter combination identified by the GWO algorithm. Supervise the training of the model using the training set. During training, implement an early stopping mechanism to prevent over-fitting, and record the training loss and validation accuracy curves to monitor the training progress.

Step 6: Input the test set into the trained GRU classifier, compute the classification accuracy of the model for each fault category, and assess the diagnostic efficiency and generalization capability of the proposed method.

## 4. Experimental Verification

### 4.1. Experimental Description

**Figure 3:**
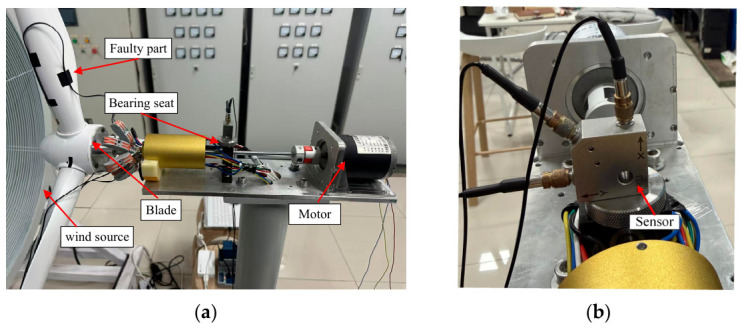
Simulation experiment setup for wind turbine blade failure. (**a**) Double fed wind turbine scaled-down test bench; (**b**) Three directional vibration sensor.

**Figure 4:**
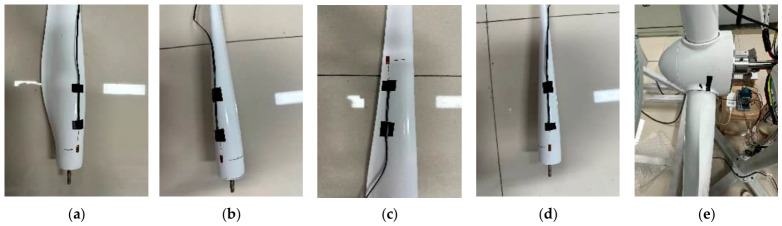
Image of prefabricated fault components. (**a**) Crack (F2); (**b**) Crack (F3); (**c**) Crack (F4); (**d**) Crack (F5); (**e**) Aerodynamic imbalance (F6).

Cracks (encompassing various locations and varying degrees of damage) and aerodynamic imbalance are two quintessential and highly detrimental failure modes in wind turbine blades [48,49]. This research utilizes the double-fed wind turbine scaled-down test bench at the National Key Laboratory for Offshore Wind Power Equipment and High-Efficiency Utilization of Wind Energy (Figure 3a) to conduct fault simulation experiments. The prefabricated fault components and their corresponding identification numbers are illustrated in Figure 4 and detailed in Table 1. Among these, the crack fault is simulated by pre-making scratches on the surface of the test blade, whereas the aerodynamic imbalance fault is induced by adjusting the installation angle of the blade. During the experiment, a CT1010LS piezoelectric triaxial IEPE vibration acceleration sensor is mounted on the blade root bearing housing (Figure 3b). The data acquisition system employs a NI-9263 32-channel, 16-bit high-speed data acquisition card, with the data acquisition software developed based on LabView 2025. The sampling frequency for vibration signals is configured at 10 kHz.

During the experimental process, the rotational frequency of the blower was set to 40 Hz. For each fault mode, five independent and replicated trials were carried out, with each trial lasting 100 s. The acquired data were divided into 1 s segments (equivalent to 10,000 sampling points), from which 1024 consecutive data points were randomly selected to form an individual sample. Each fault mode yielded 500 sample sets, collectively constituting the experimental sample dataset. This dataset was then randomly partitioned into a training set and a test set at a 7:3 ratio, implying that 70% (350 samples) from each fault mode were randomly chosen for training purposes, while the remaining 30% (150 samples) were allocated for testing.

**Figure 5:**
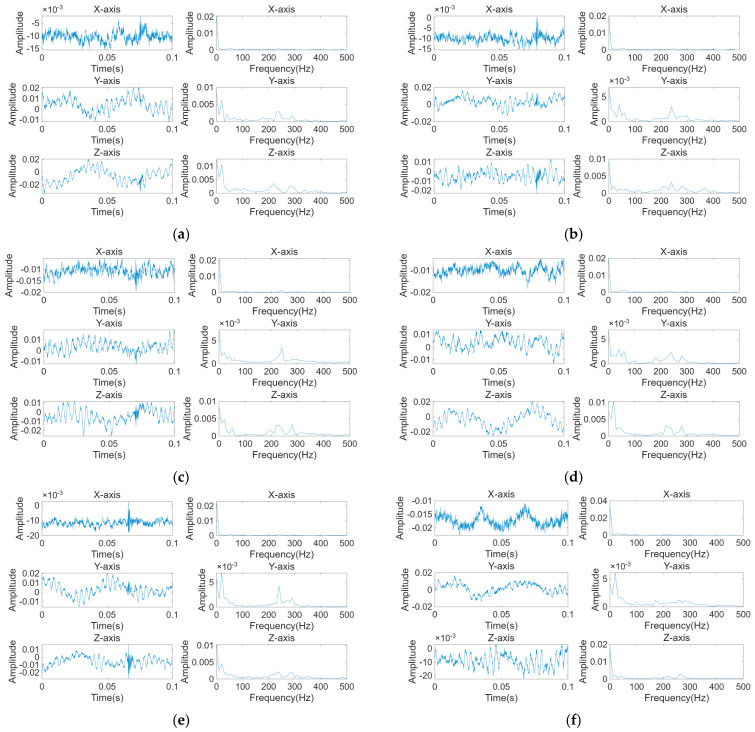
The time and frequency domain waveforms of the vibration signals. (**a**) F1; (**b**) F2; (**c**) F3; (**d**) F4; (**e**) F5; (**f**) F6.

Figure 5 illustrates the vibration time-domain waveform and its corresponding frequency spectrum derived from experimental data.

As can be discerned from the analysis of Figure 5, over extended time scales, the vibration responses associated with different fault modes exhibit discernible macroscopic characteristics; however, they are challenging to effectively distinguish within short time windows. This difficulty arises because the vibration at the blade root bearing seat is subject to multi-path transmission and structural coupling effects. The alteration in local stiffness due to minor cracks is minimal, and the energy from short-term excitation predominantly concentrates in the low-frequency fundamental and its harmonic components, resulting in similar time-domain waveforms. Frequency domain analysis indicates that the spectral energy of the X-axis across various fault modes is almost exclusively confined to the low-frequency range below 100 Hz, with negligible differences between faults and a lack of distinct pattern separability. This is attributed to the fact that stiffness in the X-axis direction is primarily governed by overall bending stiffness and centrifugal stiffening effects, rendering it difficult for cracks to significantly influence its low-frequency transmission properties. Conversely, the frequency spectra of the Y-axis and Z-axis display harmonic components induced by faults, with notable variations in amplitude, order, and distribution among different faults, offering some basis for fault identification, albeit insufficient to sustain a robust identification model. This discrepancy is primarily because the Y-axis and Z-axis are more responsive to asymmetric and nonlinear stiffness variations, which readily provoke harmonic responses. Nevertheless, factors such as sensor placement, structural connection complexity, system damping effects, environmental noise, and multi-fault interactions collectively contribute to the relatively weak harmonic energy, thereby substantially limiting pattern separability.

### 4.2. Feature Extraction and Selection

#### 4.2.1. Feature Extraction

**Figure 6:**
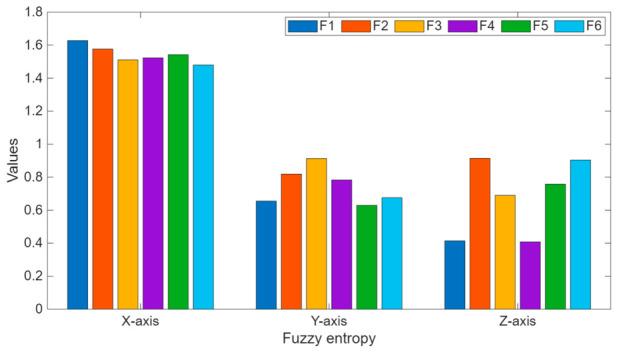
FE of the vibration signals.

**Figure 7:**
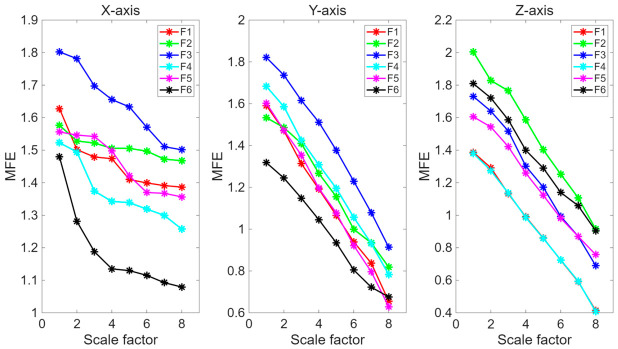
MFE of the vibration signals.

**Figure 8:**
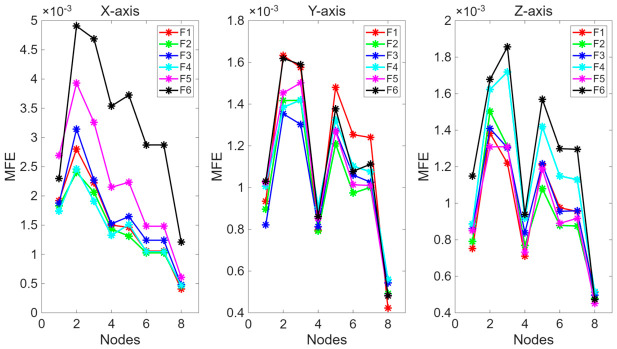
EHFE of the vibration signals.

**Figure 9:**
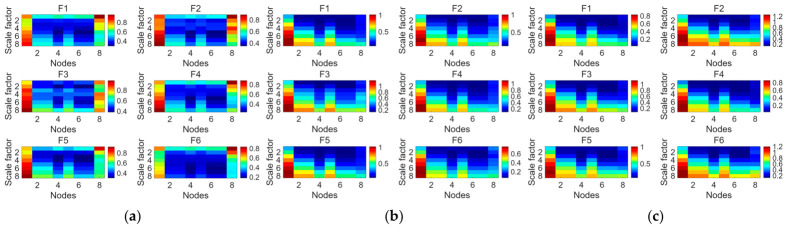
MEHFE of the vibration signals. (**a**) X-axis; (**b**) Y-axis; (**c**) Z-axis.

Matlab 2025b was used to implement the aforementioned algorithms. Based on the parameter settings listed in Table 2, the simulated experimental data were analyzed using the FE, MFE, EHFE, and MEHFE algorithms, respectively, to extract the entropy features of the vibration signals. The results are presented in Figure 6, Figure 7, Figure 8 and Figure 9.

Figure 6 depicts the FE of vibration signals. While FE exhibits sensitivity to variations in both signal amplitude and frequency, in simulation experiments, the alterations in local stiffness induced by faults are relatively subtle. The excitation energy predominantly resides in low-frequency components, resulting in negligible disparities in signal complexity across diverse fault conditions. Additionally, influenced by multi-path transmission effects and structural coupling, the capacity of FE to represent fault information is further attenuated, consequently yielding a low degree of discrimination among FE values for various fault modes.

Figure 7 depicts the MFE of vibration signal. The minimal discrepancies in MFE values among different fault modes under varying scale factors suggest that MFE exhibits insensitivity to subtle fault features arising from local stiffness alterations. Additionally, it struggles to mitigate the interference caused by the coupling effect between the transmission path and the structure, thereby failing to effectively accentuate the dynamic disparities among distinct fault modes.

Figure 8 illustrates the EHFE of the vibration signal. The variation trend of EHFE values with node number under different fault modes demonstrates a high degree of consistency, with minimal numerical differences observed between distinct fault modes. Notably, on the Y-axis and Z-axis, multiple curves almost entirely coincide. This implies that EHFE still exhibits insufficient sensitivity to the subtle fault characteristics induced by local stiffness changes, rendering it challenging to amplify the dynamic differences between various fault modes. Consequently, the ability of fault characteristics to be expressed in the entropy domain is constrained.

In contrast, the MEHFE depicted in Figure 9 markedly ameliorates the aforementioned problems. The MEHFE values for different fault modes demonstrate distinct and consistent variations with changes in scale factors and node numbers, indicating excellent separability. This suggests that through the integration of multi-scale coarse granulation and enhanced hierarchical decomposition, MEHFE can effectively preserve fault-related information across various scales while mitigating the interference of transfer paths and structural coupling effects on feature representation. Additionally, MEHFE exhibits heightened sensitivity to subtle fault features induced by local stiffness variations, allowing it to accentuate the dynamic discrepancies among different fault modes. Consequently, MEHFE can effectively address the limitations of traditional entropy features in fault discrimination and enhance the precision of pattern recognition.

**Figure 10:**
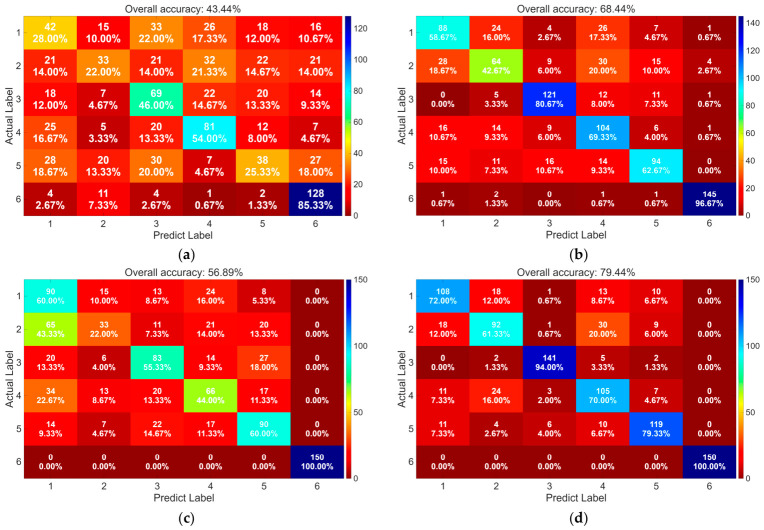
Classification confusion matrix of different entropy + GRU network. (**a**) FE+GRU; (**b**) MFE+GRU; (**c**) EHFE+GRU; (**d**) MEHFE+GRU.

To validate the efficacy of MEHFE in improving the accuracy of fault diagnosis for wind turbine blades, the extracted entropy features were fed into a GRU classifier comprising two hidden layers and one fully connected layer. The network hyper-parameters were configured as presented in Table 3 [50]. For each combined model integrating entropy features with the GRU network, 20 independent training and testing iterations were carried out. Throughout the training phase, the batch size was established at 32, and the training spanned 50 epochs. Table 4 displays the mean diagnostic accuracy rates, standard deviations, and confidence intervals for each combined model, while Figure 10 depicts the optimal classification confusion matrix derived from the 20 independent diagnostic trials.

As evident from Table 4, the MEHFE+GRU combination demonstrates the highest mean accuracy. Not only is its confidence interval markedly higher than those of the remaining three models, but it also features the narrowest interval width and the lowest standard deviation, highlighting distinct advantages in both classification performance and stability. This suggests that MEHFE exhibits exceptional discriminative capability and robustness in fault diagnosis, with its overall performance significantly outperforming that of the entropy features FE, MFE, and EHFE.

To objectively assess the statistical significance of the performance advantage of MEHFE+GRU over the three comparative models, an independent-samples *t*-test (α=0.05) was performed, using MEHFE+GRU as the reference model and comparing it separately with FE+GRU, MFE+GRU, and EHFE+GRU. The results indicated that the *p*-values for comparisons between MEHFE+GRU and each of the comparative models were all approaching zero (p<<0.001), substantially below the significance threshold. This demonstrates that MEHFE+GRU exhibits significantly higher diagnostic accuracy than the three comparative models, with the advantage being statistically robust.

Figure 10 further elucidates the fault classification performance of various entropy features. Specifically, FE solely computes the single fuzzy entropy of the original signal, leading to the lowest accuracy due to the scarcity of information. Although EHFE can explore the complexity of different frequency levels within the signal through high-low frequency decomposition, its analysis remains confined to the original time scale, resulting in the loss of cross-scale dynamic information and consequently limiting its performance. In contrast, MFE extracts complexity features of the signal at various time resolutions through multi-scale coarse granulation, providing a more comprehensive reflection of the signal’s dynamic changes and thereby achieving higher accuracy. MEHFE, however, integrates the respective strengths of multi-scale and hierarchical analysis, taking into account both variations in time scale and thoroughly exploring complexity patterns of different frequency components. This approach enables the construction of the most comprehensive and discriminative feature set, ultimately yielding the highest classification accuracy.

#### 4.2.2. Feature Selection

**Figure 11:**
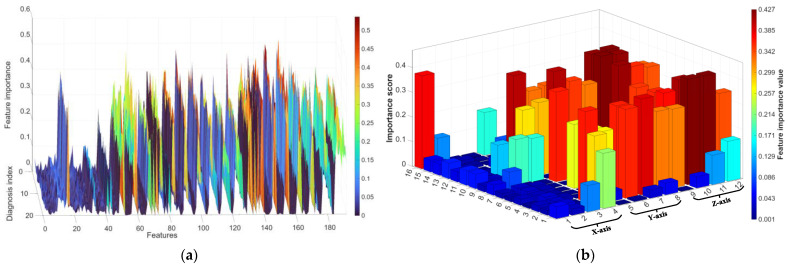
Feature importance evaluation results. (**a**) Waterfall chart of 20 test results; (**b**) Histogram of importance mean values.

In Table 4 and Figure 10, the MEHFE+GRU model achieves an average accuracy of 77.64% and a maximum accuracy of 79.44%. This performance can be attributed to the fact that the input to the GRU network comprises 192-dimensional MEHFE features derived from vibration signals in three directions, which inevitably introduces substantial information redundancy and noise interference, thereby compromising the efficiency of fault identification and classification. To mitigate this issue, an Isolation Forest model is employed for feature selection through feature importance evaluation, aiming to eliminate redundant features and enhance diagnostic efficiency. The algorithm parameters are configured as follows: the number of isolation trees is set to 200, and the sub-sampling size is 256. To ensure the robustness of the evaluation results, the algorithm is executed independently 20 times. The importance evaluation results for the 192 features are illustrated in Figure 11.

Analyzing Figure 11, it can be seen that the features with higher importance are mainly concentrated in the Y-axis and Z-axis. This phenomenon is consistent with the physical sensitivity rules of the original vibration signals and entropy features: the X-axis is dominated by overall bending stiffness and centrifugal stiffening effects, and is insensitive to local stiffness changes. Its entropy values are highly overlapped under various fault modes, with low differentiation; in contrast, the Y-axis and Z-axis are more sensitive to blade asymmetry, nonlinear stiffness changes, and aerodynamic loads, and can effectively carry fault-related information. Their MEHFE features exhibit significant differences between different fault modes. The above feature importance assessment results further verify the dominant role of Y-axis and Z-axis vibration features in fault diagnosis from the perspective of feature selection.

**Figure 12:**
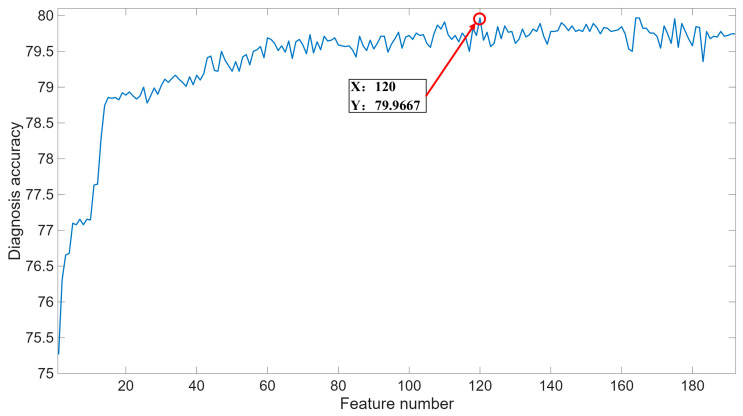
Impact of different numerical features on diagnostic accuracy.

To select an optimal number of features for constructing the best feature subset, the 192-dimensional features of MEHFE were ranked in descending order of significance. Following this, feature dimensions were incrementally added and fed into the GRU network for both training and testing. Figure 12 demonstrates the influence of varying feature quantities on the accuracy of fault diagnosis within the test sets. As observed in the figure, when the number of input features is limited, the GRU network struggles to acquire adequate information for effective fault classification, leading to subpar accuracy. However, as the feature count gradually rises, accuracy improves correspondingly, peaking at 79.96% when the feature number reaches 120. Subsequently, any further increase in features results in a decline in accuracy, which then stabilizes, suggesting that redundant feature information adversely affects the model’s classification performance.

### 4.3. Fault Diagnosis Analysis

#### 4.3.1. Fault Diagnosis Utilizing GWO-GRU

**Figure 13:**
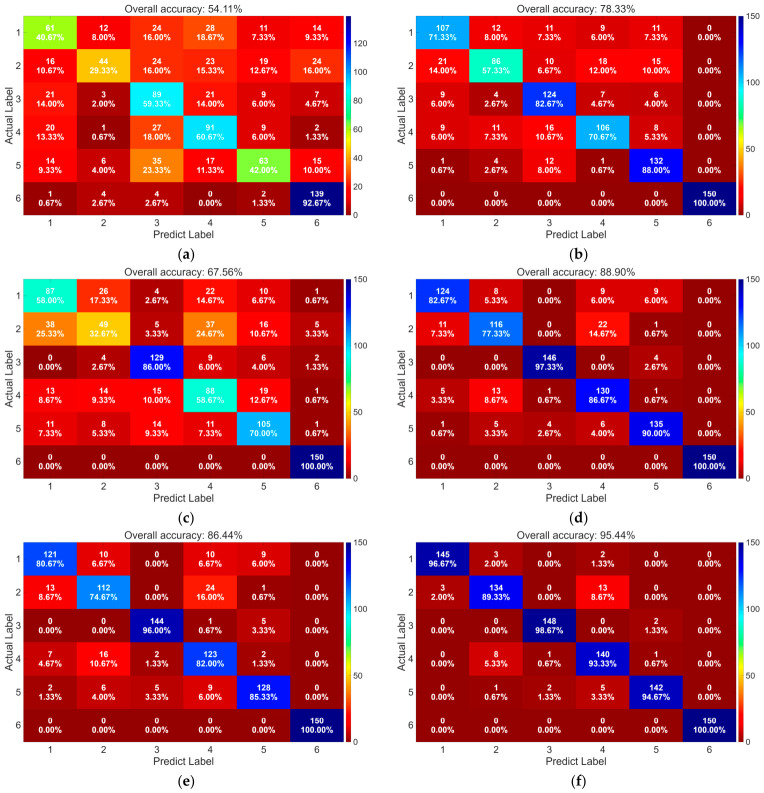
Classification confusion matrix of combined models. (**a**) FE+GWO-GRU; (**b**) MFE+GWO-GRU; (**c**) EHFE+GWO-GRU; (**d**) MEHFE+GWO-GRU; (**e**) MEHFE+IF+GRU; (**f**) MEHFE+IF+GWO-GRU.

Based on the analysis results depicted in Figure 12, the top 120-dimensional features with the highest significance in MEHFE were selected to constitute the optimal feature subset. To further enhance the precision of fault diagnosis, the GWO algorithm was utilized for adaptive optimization of the network hyper-parameters outlined in Table 3. The gray wolf population size was configured to 5, and the maximum number of iterations was set at 8. The optimization scope is detailed in Table 5. Subsequently, each of the six combined models listed in Table 6 underwent 20 independent training and testing iterations. The optimal network hyper-parameters for each combined model are presented in Table 7, while the mean, standard deviation and confidence interval derived from the 20 independent diagnostic runs are displayed in Table 8, and the optimal classification confusion matrix is illustrated in Figure 13. The results of the independent samples *t*-test (α=0.05), with MEHFE+IF+GWO-GRU as the reference model, indicate that the *p*-values for comparisons between this model and the other five models are all approaching zero (p<<0.001), thereby demonstrating the statistical robustness of its superior diagnostic performance.

A comparative analysis of Table 4 and Table 8, along with Figure 10 and Figure 13, reveals that the GRU network, with hyper-parameters configured based on empirical settings, is prone to under-fitting or over-fitting when processing feature inputs from diverse distributions. This susceptibility arises from the incompatibility between the hyper-parameters and the inherent data characteristics, leading to unsatisfactory classification performance and generalization capabilities. Nevertheless, the integration of GWO for adaptive hyper-parameter optimization enables the model to automatically seek out the optimal parameter combination, guided by the validation set accuracy. This adaptive mechanism empowers the GRU classifier to dynamically tailor its network architecture to the statistical properties of each feature set, thereby enhancing its feature mapping prowess. As a result, it not only substantially improves classification accuracy but also fortifies the model’s stability across various training-testing partitions.

Furthermore, concerning MEHFE, which is imbued with the most abundant information, its high-dimensional attributes inevitably encompass redundant and noisy elements. Directly feeding these features into a classifier engenders the risk of over-fitting and obscures the pivotal discriminative information. To tackle this challenge, the Isolation Forest algorithm is harnessed to evaluate the significance of each dimensional feature and to construct an optimal feature subset. This strategy effectively purges irrelevant or disruptive features while preserving the most discriminative core information. This feature selection procedure markedly mitigates the over-fitting propensity of the GRU model, thereby further elevating classification accuracy.

By integrating MEHFE feature selection with GWO-GRU hyper-parameter optimization in a sequential manner, a comprehensive diagnostic chain is established, encompassing “feature extraction—feature selection—parameter optimization—pattern recognition”. Feature selection furnishes the classifier model with input features of high quality and low redundancy, whereas GWO tailors the most appropriate hyper-parameters to the GRU network architecture. The collaborative effect of these two components substantially enhances the final diagnostic accuracy in comparison to the baseline model that lacks both optimization and feature selection. The aforementioned results demonstrate that the proposed method in this research, which amalgamates MEHFE, Isolation Forest, and GWO-GRU, not only attains superior accuracy in fault classification tasks but also exhibits robust performance. This fully underscores that, in the realm of complex mechanical fault diagnosis, the joint application of feature engineering and model optimization outperforms enhancements made in isolation, thereby offering a dependable technical approach for the development of intelligent diagnostic systems.

#### 4.3.2. Noise Resistance Experiment

**Figure 14:**
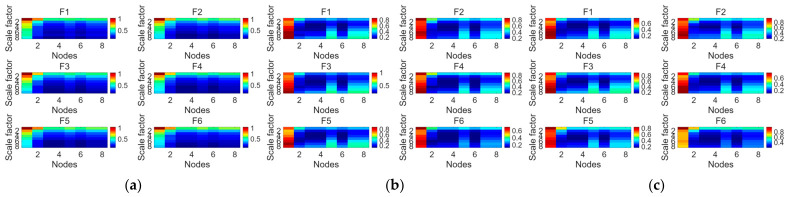
MEHFE of the vibration signals with additional white noise, SNR = 3 dB. (**a**) X-axis; (**b**) Y-axis; (**c**) Z-axis.

In practical engineering, the signals acquired frequently incorporate noise. To evaluate the anti-noise capability of the method presented in this research, 3dB Gaussian white noise was superimposed onto the experimental data. The MEHFE of the noise-contaminated signal is depicted in Figure 14.

**Figure 15:**
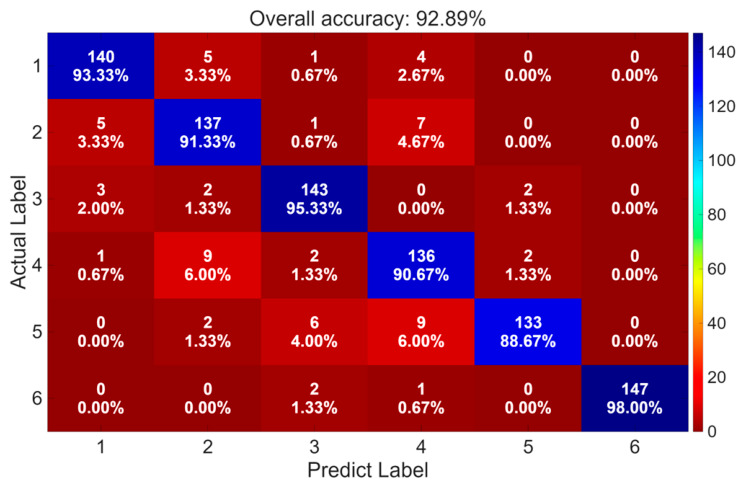
Classification confusion matrix of MEHFE+IF+GWO-GWO.

An Isolation Forest model to evaluate the significance of MEHFE entropy features was constructed, and the top 120 most significant features were selected to constitute the optimal feature subset, which was subsequently fed into the GRU network. The Grey Wolf Optimizer (GWO) was utilized to adaptively tune the network hyperparameters. Following 20 independent training and testing iterations, the mean accuracy attained 91.79%, with a standard deviation of 0.82% and a 95% confidence interval spanning [91.41%, 92.17%]. The confusion matrix illustrating the optimal classification outcomes is depicted in Figure 15. The test results demonstrate that, even under noisy conditions, our proposed method preserves high accuracy and robustness, exhibiting strong resistance to noise.

## 5. Conclusions and Prospects

Optimizing feature engineering to extract effective fault features from complex vibration signals is the key to achieving efficient fault diagnosis of wind turbine blades. This research proposes a diagnostic method that integrates MEHFE, Isolation Forest, and GWO-GRU, and validates it through fault simulation experiments. The following conclusions are drawn:

(1) MEHFE combines multi-scale coarse granulation with multi-level high-low frequency decomposition, enabling a two-dimensional joint analysis of vibration signals in the “scale-frequency” domain. Utilizing the same GRU classifier, MEHFE achieves a diagnostic accuracy of 77.64%, significantly outperforming FE (39.88%), EHFE (54.85%), and MFE (66.83%), while also exhibiting the lowest standard deviation (0.82%). This demonstrates its capability to extract comprehensive and highly discriminative fault features.
(2) Isolation Forest was utilized to evaluate the significance of the 192-dimensional MEHFE features and to select an optimal 120-dimensional subset, effectively removing redundancy and noise. This resulted in an accuracy enhancement from 77.64% to 85.52%, along with a 37.5% reduction in feature dimensionality, thereby diminishing the risk of overfitting.
(3) By employing the selected optimal feature subset as input to the GRU classifier and introducing Grey Wolf Optimizer (GWO) for adaptive optimization of GRU hyperparameters, the diagnostic accuracy was further elevated to 94.34%, with a concurrent decrease in standard deviation to 0.76%. Even under the condition of 3dB Gaussian white noise, the accuracy remained at 91.79%. The synergistic impact of feature selection and hyperparameter optimization led to a 54.46 percentage point improvement in overall accuracy compared to the baseline model (FE-GRU), underscoring the efficacy and robustness of this approach in fault diagnosis.

Future research will concentrate on the following aspects: Firstly, adaptive methods will be introduced to dynamically ascertain parameters within MEHFE, including the scale factor, decomposition layers, and similarity tolerance, based on the complexity of the signal. Secondly, SHAP value analysis or embedded sparse learning will be integrated to further delve into the interactions among features, thereby optimizing feature selection. Thirdly, GRU will be substituted with other representative deep learning networks, such as XGBoost, CNN, and Transformer, to validate the generalization capability and applicable scope of the MEHFE method across diverse classifier architectures. Fourthly, lightweight techniques like knowledge distillation and model pruning will be explored to facilitate the deployment of the model on edge computing devices, enabling real-time fault diagnosis of wind turbine blades and advancing the engineering implementation of the proposed method.

## Figures and Tables

**Table 1:** Description of prefabricated fault components.

Label	Failure Mode	Location	Degree	Image
F1	Healthy			
F2	Crack	Windward blade root	The crack length is 50% of the local load path	Figure 4a
F3	Crack	Windward blade root	The crack length is 75% of the local load path	Figure 4b
F4	Crack	Middle part of the windward leaf	The crack length is 50% of the local load path	Figure 4c
F5	Crack	Leeward side blade root	The crack length is 70% of the local load path	Figure 4d
F6	Aerodynamic imbalance		The blade is rotated clockwise with 5°	Figure 4e

**Table 2:** Parameter settings for entropy calculation methods.

Parameters	Values
Gradient of the fuzzy function n	2
Embedding dimension m	2
Similarity tolerance r	0.2 SD
Scale factor τ	8
Analysis level k	3

**Table 3:** Parameter settings for GRU network.

Parameters	Values
Number of neurons in hidden layer	128
Number of neurons in fully connected layer	64
Dropout rate	0.1
Learning rate	0.001

**Table 4:** Diagnostic results of different entropy + GRU network.

Models	Mean Accuracy (%)	Standard Deviation (%)	95% Confidence Interval (%)
FE+GRU	39.88	2.51	[38.71, 41.05]
MFE+GRU	66.83	0.99	[66.37, 67.29]
EHFE+GRU	54.85	2.31	[54.77, 56.93]
MEHFE+GRU	77.64	0.82	[77.26, 78.02]

**Table 5:** Optimization range of GRU network’s hyper-parameters.

Parameters	Ranges
Number of neurons in hidden layer	16~256
Number of neurons in fully connected layer	16~128
Dropout rate	0.0~0.5
Learning rate	0.0001~0.01

**Table 6:** Combined models for comparative analysis.

Models	Description
FE+GWO-GRU	FE is the input feature, and GRU is optimized using GWO
MFE+GWO-GRU	MFE is the input feature, and GRU is optimized using GWO
EHFE+GWO-GRU	EHFE is the input feature, and GRU is optimized using GWO
MEHFE+GWO-GRU	Take all MEHFE features as input, and GRU is optimized using GWO
MEHFE+IF+GRU	Take the 120 most important MEHFE features as input, and GRU is not optimized
MEHFE+IF+GWO-GRU	Take the 120 most important MEHFE features as input, and GRU is optimized using GWO

**Table 7:** Optimal GRU network’s hyper-parameters.

Parameters	FE+ GWO-GRU	MFE+ GWO-GRU	EHFE+ GWO-GRU	MEHFE+ GWO-GRU	MEHFE+IF+GWO-GRU
Number of neurons in hidden layer	178	114	136	150	153
Number of neurons in fully connected layer	122	74	73	70	60
Dropout rate	0.356	0.042	0.338	0.291	0.302
Learning rate	0.0013	0.0005	0.0007	0.0007	0.0007

**Table 8:** Diagnostic results of combined models.

Models	Mean Accuracy (%)	Standard Deviation (%)	95% Confidence Interval (%)
FE+GWO-GRU	51.86	2.03	[50.91, 52.81]
MFE+GWO-GRU	76.98	0.90	[76.56, 77.40]
EHFE+GWO-GRU	65.43	1.83	[64.57, 66.29]
MEHFE+GWO-GRU	87.96	0.80	[87.59, 88.33]
MEHFE+IF+GRU	85.52	0.77	[85.16, 85.88]
MEHFE+IF+GWO-GRU	94.34	0.76	[93.98, 94.70]

## Data Availability

The data that support the findings of this research are available upon reasonable request from the authors.
